# Community-Based Pharmacy Practice Innovation and the Role of the Community-Based Pharmacist Practitioner in the United States

**DOI:** 10.3390/pharmacy7030106

**Published:** 2019-08-04

**Authors:** Jean-Venable Goode, James Owen, Alexis Page, Sharon Gatewood

**Affiliations:** 1 Department of Pharmacotherapy and Outcomes Science, Virginia Commonwealth University, Richmond, VA 23298, USA; 2 Practice and Science Affairs, American Pharmacists Association, Washington, DC 20037, USA

**Keywords:** community-based pharmacy, community-based pharmacist practitioners

## Abstract

Community-based pharmacy practice is evolving from a focus on product preparation and dispensing to becoming a health care destination within the four walls of the traditional community-based pharmacy. Furthermore, community-based pharmacy practice is expanding beyond the four walls of the traditional community-based pharmacy to provide care to patients where they need it. Pharmacists involved in this transition are community-based pharmacist practitioners who are primarily involved in leading and advancing team-based patient care services in communities to improve the patient health. This paper will review community-based pharmacy practice innovations and the role of the community-based pharmacist practitioner in the United States.

## 1. Introduction

### 1.1. An Overview of Health Care and the Role of Community-Based Pharmacists

There are substantial challenges related to public health including issues associated with health care inequalities, aging populations, increasing levels of chronic disease and urbanization [1]. There is a need to increase access to primary care services, control costs, and improve outcomes in health care for patients especially in the management of chronic conditions which puts a strain on health care systems worldwide [2]. Addressing these issues is critical for improving the health of patients in communities. To fully address these issues, all types of health care providers with the necessary knowledge, skills, abilities, and required competencies must be utilized whenever possible to provide needed care.

From a global perspective, community-based pharmacists represent the third largest health care professional group outnumbered only by physicians and nurses [2]. Community-based pharmacists are an underutilized health care provider who can improve access to care when and where patients want to receive it. Fortunately, a trend is emerging for community-based pharmacists to function as care extenders to counter primary care provider shortages and address the substantial problems and associated costs due to the inappropriate use of medication [2]. While the types of patient care services that community-based pharmacists are providing are extremely variable by country or even by provincial or state jurisdiction, more and more pharmacists are providing important emergency medication refills, renewals/extensions of prescriptions, changes to doses or formulations, therapeutic substitution, prescribing for minor ailments, initiation of prescription drug therapy, ordering and interpreting laboratory tests, and administering drugs by injection [2].

In the United States, the challenges to the health care system continue to mount. Despite changes to health care laws and coverage intended to insure more individuals and decrease costs, a substantial portion of the population remains without insurance coverage. Additionally, health care cost continues to be a significant portion of the gross domestic product in the United States (US) and is projected to grow by 5.5% from 2018–2027 [3]. Prescription drug costs are expected to increase an average of 6.1% per year during the same time due to the introduction of new drugs and a focused effort for patients with chronic disease to adhere to their medication regimen [3]. Pharmacists, who practice in community-based settings are key to improving adherence to prescribed medications. As the US health care system continues to evolve, a primary focus is on outcomes and quality as ways to manage costs effectively and efficiently [4].

Currently, there are shortages of health care providers with increasing shortages in primary care predicted for the future [4]. Specifically for physicians, it is predicted there will be a shortage of at least 43,000 primary care physicians and 140,000 total physicians in the US by 2030 [5]. Community-based pharmacists, however, can help to address this problem. The US Bureau of Labor Statistics estimates that there are more than 186,000 community-based pharmacists in the United States [6]. Increasingly, the pharmacists’ role is being recognized as an important as the US health care environment changes and in initiatives to reform healthcare [7].

### 1.2. Defining Community-Based Pharmacist Practitioners

Community-based pharmacy settings positively impact patient care as a result of their convenience and as supported by the frequency of access by patients. One study revealed patients visited a community-based pharmacy 35 times per year compared with a primary care physician only four times per year [8]. Additionally, pharmacists are available in these settings with 58% of employed pharmacists in traditional community-based pharmacy settings (pharmacy, supermarket or general merchandise) [6]. Other community-based pharmacies include health-systems, Federally-Qualified Healthcare Centers, clinics, and specialty. Pharmacists are also in community-based settings beyond the traditional “four walls” of a pharmacy including physician offices, patient homes, churches, and work places [9,10].

Pharmacists practicing in community-based settings are health care providers who offer either generalist or specialist ambulatory care services to patients in the communities they serve [9]. The primary goal of a community-based pharmacist practitioner is to keep patients healthy [10]. Community-based pharmacist practitioners create, advance, and influence team-based care; strive to enhance management of community-based pharmacy practices to focus on the delivery of patient care services; serve as leaders within community-based pharmacy settings, local communities, and the profession of pharmacy; and provide direct patient care to meet the healthcare needs of the communities that they serve [9]. Regardless of the actual physical practice setting location, the focus of the community-based pharmacist practitioner is providing patients with the care they need, when and where they need it.

Community-based pharmacist practitioners provide a wide range of services including educational consultations, medication management and other medication optimization services, chronic condition management, patient empowerment, care coordination, health and wellness services, and other services that help to improve the lives of patients in the community [9]. Community-based pharmacist practitioners are essential health care professionals who provide direct patient care, advance team-based care, manage services that focus on the patient, and serve as leaders within their communities and the profession [9]. As a greater focus is placed on the importance of chronic disease management, wellness, and medication management the opportunities for community-based pharmacist practitioners are expected to continue to grow [11].

### 1.3. Challenges Facing Community-Based Pharmacist Practitioners in the United States

The underlying issues surrounding the expansion of community-based pharmacists’ role in patient care no matter the country requires dedicated remuneration, primary care integration, and multidisciplinary education [2]. In traditional community-based pharmacies, there are multiple challenges for community-based pharmacist practitioners implementing innovative patient care services. The physical layout of the traditional community-based pharmacy does not facilitate patient care services. Many community-based pharmacist practitioners are adding semi-private counseling areas, private counseling rooms, exam rooms, and conference rooms for innovative patient care service delivery. The workflow process in community-based pharmacies can also be a challenge. The process for dispensing of prescription medications usually requires the pharmacist as the person responsible for drug utilization review and the verification of the prescription. The number of support staff can greatly impact the pharmacist’s ability to be involved with other patient care services along with dispensing of product. Several methods have been used to overcome the issue of time for the pharmacist to provide patient care services including adding more support staff, pharmacist overlap (i.e., more than one pharmacist working at a time) and using a technician checking technician, if allowed by state law [12,13].

It is essential to change, update, and redesign the business model in community-based pharmacy practice. The focus of community-based pharmacist practitioner activities need to be on patient-centered care to maximize their impact in the communities they serve. Schommer and colleagues state that you must “create payment and business models for community-based pharmacy practice, advance pharmacy technician practice, expand community-based pharmacy residency programs, and begin seeing transformations through the patient’s eyes” [14]. This fundamental paradigm shift to the business model of community-based pharmacy practices will establish the pharmacy practice location as a heath care access point where the practice is reimbursed for the public health services they are providing [14]. The redesign of community pharmacy practices as settings of care is essential including changing the business model, implementing strategies such as medication synchronization, integrating technology and maximizing technicians [15].

Another challenge for community-based pharmacist practitioners is integrating technology in community-based practices. Currently, the majority of community-based pharmacies use a dispensing system with or without the capability to document patient care services, limiting the ability to document services. Furthermore, the pharmacy systems usually do not integrate with the electronic health care record (EHR). Several recent articles have been published documenting the value of EHR integration [16,17]. There is also a movement for documentation and patient care services through the use of a pharmacist e-care plan [18]. New systems enabling standards and technology systems will hopefully help to address these issues primarily focusing on consistent electronic documentation methods and strategies for consistently coding and documenting care provided to patients [19].

Additionally, the location of community-based pharmacy practices can be a barrier to the provision of team-based care and integration [20]. However, as the community-based pharmacist practitioner expands beyond the four walls of the traditional based-community pharmacy it will become easier to engage in team-based care. Furthermore, the profession does not have a referral process that is similar to other health care providers. There needs to be process for referrals between pharmacists and other healthcare providers as well as other pharmacists. Integration of technology may help with team-based care and the referral process.

Expanding the role of pharmacists where they are practicing at the “top of their license” to improve care and drug therapy outcomes is essential to the activities of a community-based pharmacist practitioner and has been promoted by the World Health Organization [7,20]. In 2007, Canada enacted laws that enabled expanded scope in some provinces through the Additional Prescribing Authorization (APA) [20]. In the United States, the practice of pharmacy is regulated by individual state boards of pharmacy and scope of practice and authority varies greatly from state to state. States such as Oregon, California, Idaho, and Washington have begun to expand the scope of practice and authority for pharmacists to prescribe medications, perform point of care diagnostic tests, initiate hormonal contraception, and administer injectable medications [21]. However, continued expansion of changes remains a challenge in many states, but important to allow community-based pharmacist practitioners to provide the needed patient care services in the communities they serve.

A major obstacle for community-based pharmacists in the United States is that pharmacists lack formal designation as providers in the federal Medicare program. Specifically, pharmacists are not recognized as providers under Section 1861(s) (2) of the Social Security Act, as such pharmacists are not paid by the Federal government for health care services under the Medicare Part B program. To address this important barrier, professional associations, and other stakeholders have advocated for decades that action be taken through the US legislative process to include pharmacists and enable a payment mechanism for the services that pharmacists can provide to patients. It is important because other payers in the US typically follow Medicare payment policies, thus limiting the ability of pharmacists from receiving payment from these other public, private and commercial entities. The reason that recognition in the Federal Medicare program is viewed by many as the key to the establishment of successful payment models nationwide. However, it is important to note that many states have designated pharmacists as providers, however, this recognition may or may not guarantee payment for service. Pharmacist services need recognition as being valuable by patients and other payers [7].

While the overall number of potential community-based pharmacist practitioners is large; engaging and developing them as providers of care remains a challenge. There is a need for these individuals to undergo continuing professional development and training with a focus as on patient-centered, team-oriented, evidence-based care providers of care [22]. Additionally, there are needs for the credentialing and privileging of community-based pharmacist practitioners which facilitates provision of care and payment for service. Finally, there is an inherent difficulty in establishing value and attributing the outcomes to the pharmacist-provided patient care services [19]. What are the innovative community-based pharmacy services in the United States and the role of the community-based pharmacist practitioner? 

## 2. Methods

This review was developed based on the author’s knowledge of community-based pharmacy practice. The authors define the service delivery model and innovative services and provide support from the literature where available. The authors have developed a system for organizing the types of community-based pharmacy innovative patient care services characterizing all of the patient care services in six different categories. All of the types of innovative patient care services are incorporated in one of the six categories. Additionally, the authors describe the services with a focus on the role of the community-based pharmacist practitioner [9].

## 3. Findings

The findings include a description of community-based pharmacy practice innovations and the role of the community-based pharmacist practitioner in the United States. First, the service models are described to provide insight on the framework for the delivery of the patient care in community-based pharmacy practice. Then the types of the innovative patient care services are described with detail under the six categories. It is important to note that all of the types and categories of patient care services may be offered within the different service delivery models. Most of the innovative patient care services are supported by recent literature. Furthermore, a brief global context is offered about the impact of innovations in the US.

### 3.1. Models of Community-Based Patient Care Service Delivery

Traditionally, around the world, community pharmacists have been viewed as a distributor of medications [1]. This, however, is shifting as now with the public viewing pharmacists as health care providers. To support this, research has been published that indicates patient and pharmacist preferences for care provided in community-based pharmacies [23]. Published in this research, patients report that from their perspective, the optimal service model includes options for appointments with a health care provider in the pharmacy, having access to the full medical record, providing a point of care diagnostic testing, offering preventive health screening, limited physical examinations, and prescribing of medications [23]. As the profession of pharmacy continues to evolve to focus on patient care, a shift is occurring from product centered to a patient-centered model of care [14]. In the US, patient’s medication experience includes both a social and personal experience [14]. Responding to these changes, the pharmacy profession has entered a new “patient-centered medication experience” era and pharmacy practices are employing principles of “collaboration theory” to implement new systems of care [14]. To address these changes, community-based pharmacist practitioners spend time and make connections to the community being visible, increasing health awareness and meeting the needs of the patients in the communities they serve [10].

**Figure 1:**
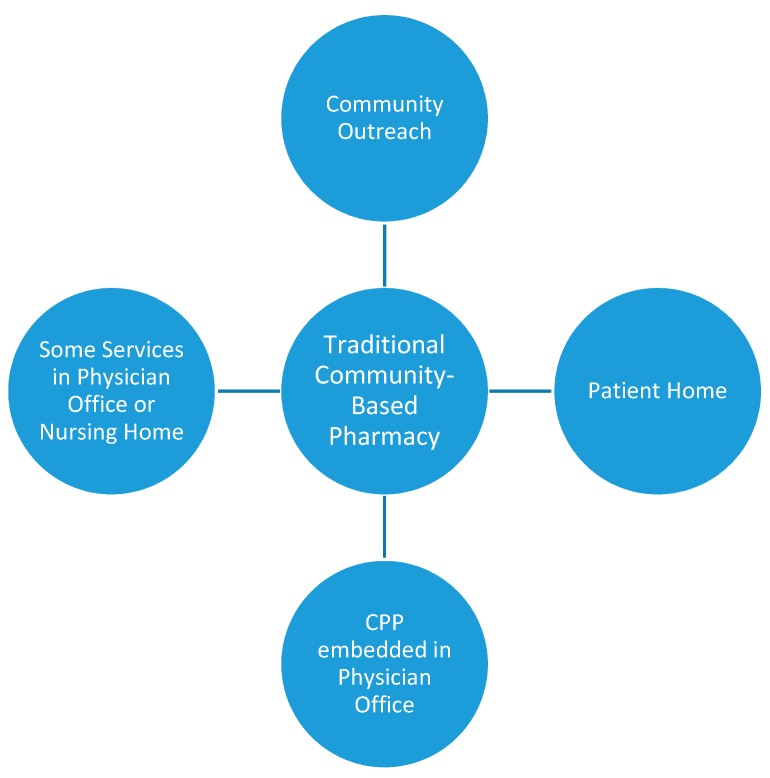
Community-based pharmacist practitioner service model.

The service model develops from the traditional community-based pharmacy or four walls and expands beyond to the community (Figure 1). Additionally, the four walls of the traditional community-based pharmacy are changing, becoming health care destinations which even include health care classes such as yoga. Community-based pharmacist practitioners are participating in outreach events in the communities, churches, workplaces, shopping malls, etc. Community-based pharmacist practitioners are conducting home visits form traditional community-based pharmacies. Community-based pharmacist practitioners are also being placed in physician offices to provide care. These patient care services are on a part-time basis where the community-based pharmacist practitioner spends some portion of their time in the physician office or the community-based pharmacist practitioner is embedded in the office on a full-time basis, but employed by the traditional community-based pharmacy. This article will focus on innovative community-based pharmacy services that are encompassed within this service delivery model.

### 3.2. Community-Based Pharmacy Services

Community-based pharmacist practitioners in community-based settings are providing innovative patient care services beyond preparing and dispensing prescription products. These services are categorized into the following areas: medication optimization, wellness and prevention, chronic care management, acute care management, patient education, and other services (Table 1). This manuscript will review those services and supporting literature, if available.

#### 3.2.1. Medication Optimization

Medication optimization is defined as a patient-centered, collaborative approach to managing medication therapy that is applied consistently and holistically across care settings to improve patient care and reduce overall health care costs [24]. Medication optimization services are comprehensive and include services directly related to the medication including medication packaging and home delivery and other services such appointment-based medication synchronization, other adherence programs, comprehensive and targeted medication management, and deprescribing.

Community-based pharmacists are also making an impact on adherence through appointment-based medication synchronization. This process has been shown to increase adherence and improve chronic health conditions, as well as reduce overall cost [25,26]. Although synchronization can help remind patients, provide updates on their progress, simplify the process, and make refilling a prescription more convenient, nonadherence can be multifactorial. The monthly appointments with the pharmacist are critically important, because it allows the pharmacist to educate, engage, and solve problems [26]. However, some community-based pharmacist practitioners may provide only medication synchronization without the appointment with the pharmacist as a way to just align refills and avoid trips to the pharmacy. Other adherence programs include counseling and education by pharmacists and automatic refills [27]. Community-based pharmacist practitioners are helping through medication packing and home delivery. Medication packing helps patients to take their medications correctly and know if they have missed a dose. Community-based pharmacist practitioners package patient medications according to days of the week or time of day. Another mechanism for improving patient adherence is through community-based pharmacist practitioner administration of medications such as long-acting injectables (e.g., antipsychotics, contraceptives) and vitamin B-12 [28]. Home delivery of medications has increased patient access to their medications by taking away the barriers of transportation and proximity to a pharmacy.

Medication reconciliation is the process of creating the most accurate medication list to increase patient safety, decrease medication related problems, and improve health outcomes [29,30]. One of the barriers to maintaining an accurate medication list happens when the patient is discharged from a healthcare facility to another facility because there is loss to follow up. The Affordable Care Act in 2012 launched measurement of hospitals on performance with the Hospital Readmission Reduction Program. This program is administered by the Centers for Medicare and Medicaid Services (CMS) and penalizes hospitals with excess readmissions within 30 days of discharges for certain chronic diseases [31]. Community-based pharmacist practitioners are emerging in the transitions of care area to help decrease readmissions rates and emergency room visits and increase healthcare cost savings [32,33]. Physicians in multiple settings believe that community-based pharmacists should be a part of the transitions of care process because of their medication knowledge and access to patients [34,35].

Medication management services also contribute to medication optimization. Medication management services are a spectrum of patient-centered, pharmacist-provided, collaborative services that focus on medication appropriateness, effectiveness, safety, and adherence with the goal of improving health outcomes [36]. Typically, in practice there are two different distinct services; either comprehensive medication management or targeted medication review. Comprehensive medication management is defined as the standard of care that ensures each patient’s medications (whether they are prescription, nonprescription, alternative, traditional, vitamins, or nutritional supplements) are individually assessed to determine that each medication is appropriate for the patient, effective for the medical condition, safe given the comorbidities and other medications being taken, and able to be taken by the patient as intended [37]. Several studies have documented the positive impact of community-based pharmacist practitioner engaged in comprehensive medication management [38,39,40]. Targeted medication review (TMR) assesses medication use, monitors whether any unresolved issues need attention, new drug therapy problems have arisen, or if there has been a transition in care. TMRs are also used when a potential medication therapy problem is identified and the community-based pharmacist practitioner verifies whether or not there is an actual problem and if a problem exists, an attempt is made to resolve it. Medication optimization also includes efforts by community-based pharmacist practitioners to be involved with deprescribing medications.

#### 3.2.2. Wellness and Prevention

Community-based pharmacist practitioners have been involved in wellness and prevention services through point-of-care testing (POCT) for years, including blood glucose, cholesterol, and A1c. Many states allow pharmacists to perform these laboratory tests through the state’s pharmacy practice act or by establishing a collaborative practice agreement with a provider. These services can be provided by a pharmacist once the pharmacy obtains and maintains Clinical Laboratory Improvement Amendments (CLIA) Certificate of Waiver through Centers for Medicare & Medicaid Services (CMS). The pharmacy must follow ‘good laboratory practice’ when performing tests, which address issues of proper physical environment and recording of test results with patient information in a retrievable file. The pharmacy also needs to follow Occupational Safety and Health Administration (OSHA) standards to provide a safe and healthy environment [41]. As of 2015, community pharmacies are the fourth highest-ranking entity of CLIA-waived laboratories, accounting for 5.4% of all CLIA-waived laboratory facilities [42,43]. More recently, community-based pharmacist practitioners have become involved in POCT for infectious diseases like Human Immunodeficiency Virus (HIV), Hepatitis C, streptococcus, and influenza [44,45]. As pharmacogenomics develops, POCT devices that measure drug metabolism are becoming available and community-based pharmacist practitioners with their drug expertise are a good choice to provide this service [46]. This would prevent someone from taking a medication that they cannot process and avoid having to try multiple medications to find the right one. For those states that do not allow pharmacists to provide these services, risk assessments can be provided to patients. These can determine the patient’s risk of developing or assessing the severity of conditions like depression, asthma, and cardiovascular risks. These services have been performed by pharmacists as health screenings in patients who are undiagnosed and for monitoring a patient’s chronic disease state [40].

One of the most successful services in wellness and prevention that community-based practitioners offer is immunizations [47]. Pharmacist-provided immunization services began with the administration of the influenza and pneumococcal vaccine. From there, services expanded to pharmacists administering routine adult immunizations. Pharmacist involvement with administering immunizations has increased vaccinations rates [47,48]. In 2000, pre-travel health services began to be offered to patients by community-based pharmacist practitioners [49,50]. These services not only include immunizations needed for international travel but also education, treatment, and prevention of non-vaccine preventable disease. Studies have shown that the pharmacist run pre-travel health clinics have an overall high patient satisfaction rate and recommendations are accepted by providers [51,52].

Community-based pharmacist practitioners provide tobacco cessation services and new scope of practice changes in individual state jurisdictions are facilitating the role by allowing pharmacists to prescribe medication [53,54]. Another service facilitated by practice change is the prescribing of contraceptive therapy for women [55]. Due to the increase in patient deaths from the opioid epidemic, a new role in most states is for community-based pharmacist practitioners to be involved with the dispensing and administering of naloxone. It has been shown that pharmacist involvement in the dispensing of naloxone had significant reductions in fatal opioid overdoses as opposed to just improving access [56]. Other wellness and prevention services include weight management, fluoride treatments, falls prevention, pharmacogenomics, sleep assessment, drug take back, nutraceuticals, and bioidentical hormone replacement [57,58,59,60,61,62]. Lastly, community-based pharmacist practitioners are involved in providing Medicare Annual Wellness Visits in physician offices [63].

#### 3.2.3. Chronic Care Management

Chronic care management (CCM) is defined as services aim to deliver quality patient centered care, which can assist providers in improving patient outcomes and quality metrics [64,65]. CCM aims to increase non-face-to-face interactions with patient which helps to improve the coordination of care between provider office visits. Pharmacists are able to partner with qualified health professionals to provide CCM services under general supervision of providers, including those located in federally qualified health centers (FQHCs) or rural health centers (RHCs). Studies have shown community-based pharmacist practitioners are involved with CCM visits which make an impact on health indicators, like adherence and medication safety [66,67]. In another study, pharmacists proved that they could reduce the overall drug cost and increase the quality of care through medication management [68].

Community-based pharmacist practitioners also offer chronic care management sometimes known as disease management for chronic disease such as diabetes, hypertension, hyperlipidemia, heart failure, asthma, or hepatitis C [69]. Additionally, community-based pharmacists offer anticoagulation services. These chronic care management services may or may not be offered under collaborative practice agreements [21]. Some of these services may even be offered outside of the four walls of the community pharmacy in places such as barbershops [70]. Furthermore, the community-based pharmacist practitioner can use CLIA-waived tests for monitoring chronic disease states or these tests may be offered in the pharmacy without any association management services. These tests may include pharmacogenomics tests for ensuring patients are taking appropriate therapy. Community-based pharmacists are using saliva testing for biodentical hormone replacement therapy services.

#### 3.2.4. Acute Care Management

Antibiotic resistance is one of the largest global health problems leading to extended inpatient hospitals stays, higher medical costs, and increased mortality [71]. Community-based pharmacist practitioners are well positioned to serve as gatekeepers for antibiotic prescribing due to their considerable training and knowledge of infectious disease pathophysiology, antibiotic indication, dosing, and appropriate length of treatment [72]. In addition to being competent antimicrobial stewards, community-based pharmacist practitioners’ role in curbing antimicrobial resistance is expanding to increase patient access to care through physician-led collaborative practice agreements and CLIA waived POCT [73]. In community pharmacy-based CLIA-waived testing facilities, community-based pharmacist practitioners can screen for and treat acute infectious diseases, such as influenza A/B, Group A streptococcus (GAS) and *Helicobacter pylori*, decreasing time to therapy for patients [74].

It has been reported that patients who present with influenza-like symptoms and are not tested for influenza are twice as likely to be prescribed antiviral therapy [75]. The increasing prevalence of CLIA-waived POCT and rapid diagnostic testing (RDT) in community pharmacies can help mitigate unnecessary antimicrobial prescribing. A report on midwestern pharmacies offering influenza testing showed that of the 121 patients who presented to a community-pharmacy with influenza-like symptoms and screened for influenza, 75 patients were tested, with 8 tests yielding positive results. Seven of the eight patients who tested positive received a prescription for oseltamivir and six of these seven patients reported feeling better during 24–48-h follow-up call [76]. Initiation of appropriate antiviral therapy for patients infected with influenza is time sensitive. Community-based pharmacist practitioners offer extended hours and patients can access appropriate antiviral medications quicker by seeing a pharmacist for diagnosis and treatment than when a patient is referred to another provider [77].

Analogous to influenza POCT in community-based pharmacies is group A streptococcus (GAS) POCT, which has also grown as a community-based pharmacist provided service. Community-based pharmacist practitioners need to be conscientious antimicrobial stewards because adult pharyngitis is most commonly viral, yet it has been shown that up to 75% of patients are prescribed antibiotics [78]. *Streptococcus pyogenes* is the most common bacterial cause of adult pharyngitis, accounting for up to 15% of cases, indicating that antibiotics are only warranted for roughly 15% these adult pharyngitis cases [78]. GAS POCT can be implemented alongside influenza POCT within the same community-based pharmacies using a similar collaborative care model for managing patients that was used in several midwestern pharmacies [79]. Community pharmacy-based GAS POCT was found to be more cost effective for the diagnosis and treatment than physician-based diagnosis and treatment, in addition to being more convenient for patients. [80].

Community-based pharmacist practitioners are capable of managing and prescribing for other common acute diseases such as uncomplicated urinary tract infections (UTIs) [81]. Evidence is lacking in the US, but in a prospective trial in 39 community pharmacies in Canada, community pharmacists were able to achieve symptom resolution in 88.9% of patients who presented to the pharmacy with symptoms of a UTI, with high patient satisfaction [82]. In the United Kingdom, community pharmacists were able to screen patients for UTIs and provide trimethoprim to 73% patients, with the remaining patients receiving symptomatic management. Outcomes of this study suggest that community pharmacists can provide appropriate treatment for patients with uncomplicated UTIs and increase patient access to care [83].

#### 3.2.5. Education

Community-based pharmacist practitioners are well prepared to educate patients about medications, wellness, and prevention and medical conditions. Community-based pharmacist practitioners are playing an even larger role within their community by promoting health and wellness programs. Pharmacists are coaching on healthy eating habits, assisting with smoking cessation, and combating sedentary lifestyles [84]. The Centers for Disease Control and Prevention (CDC) recognize community pharmacists’ role in preventive health care and have even called upon them to facilitate the National Diabetes Prevention Program (DPP) throughout the country [85]. Community-based pharmacist practitioners are also providing accredited diabetes education programs through either the American Diabetes Association or American Association of Diabetes Educators [86].

#### 3.2.6. Other Services

Community-based pharmacist practitioners are serving as transitions-of-care (TOC) champions when patients are discharged from inpatient settings to help minimize gaps in care, reduce hospital readmissions, and resolve medication-related problems. When community pharmacies provided medication management services within one week of hospital discharge to patients with congestive heart failure, chronic obstructive pulmonary disease, or pneumonia, the 30-day readmission rate was 13.1% less in patients compared to patients who did not receive the service [87]. Through community pharmacy-based TOC programs, patients’ risk of readmission can be decreased by 28% and 31.9% at 30 and 180 days, respectively, when pharmacists are involved in discharge counseling, medication reconciliation, and telephone follow-up [88]. Through the utilization of pharmacists during health care setting transitions for patients, community-based pharmacist practitioners are capable of improving patient outcomes and reducing hospital readmission rates. 

Teleheath is rapidly advancing, which provides increasing opportunities for community-based pharmacist practitioners to be involved in remote pharmacy operations and patient care. Virtual pharmacist services is a new innovative practice model that has been shown to improve patient outcomes and minimize costs [65,67,89]. When patients at risk for medication-related problems were referred to a telepharmacist for chronic care management at an outpatient family medicine clinic, pharmacists generated 200 interventions over a 6-month period. The physicians accepted 37.5% of the pharmacist’s recommendations, over half of which were related to medication safety [65]. Pharmacists have also been able to show effective management of hypertension and diabetes through telepharmacy by providing medication management and lifestyle modification recommendations with medication adjustments [90,91].

Community-based pharmacist practitioners collaborate with local associations and public health agencies to provide education and serve as a resource during public health emergencies. In fact, community-based pharmacist practitioners are well positioned to respond to emergencies due to their wide distribution across urban, suburban, and rural settings, and their easy access for patients. Proposed disaster-readiness roles of pharmacists include “clinical” and “other”, with the clinical category referring to ambulatory care, community-based or outpatient clinic pharmacists with strong pharmacotherapy backgrounds, working in a variety of settings during a disaster such as a shelter, hospital, clinic, or outreach site [92]. In an evaluation of community healthcare providers ability to respond to emergencies resulting from bioterrorist attacks, pharmacists scored higher (78.5%) than physicians (71.3%) and nurses (66.5%) in their ability to demonstrate creative problem solving and flexible thinking to unusual situations [93].

Some community-based pharmacist practitioners have started a new patient care service that is involves administering an intradermal injection; tuberculin skin testing. The first report of pharmacists conducting tuberculin skin testing was in 2008 in a national grocery chain pharmacy where 18 tuberculin skin tests were administered over 11 months by two pharmacists and 17 of the 18 patients returned within the allotted time for their reading. The pharmacists felt that this service was relatively simple to integrate into workflow, with each test taking less than 10 min per patient and that it was an important public service opportunity [94]. In 2011, New Mexico pharmacists became authorized to prescribe, administer, and read tuberculin skin tests. Trained pharmacists in rural and urban New Mexico administered 606 tuberculin skin tests with 578 patients having appropriate follow-up to read the test. The authors attributed the high follow-up rates at the community pharmacies to convenience and accessible locations [95].

## 4. Innovation in the US and Global Impact

It is beyond the scope of this paper to provide a comprehensive review of global innovative community-based pharmacy practice. However, it is important to note that there is a global shift in community-based pharmacy from a dispensing retail to a health care provider practice [2]. No country has been able to transform community-based pharmacy practice completely as a sustainable health care destination without the product driving the business model. However, some countries like the US have made substantial progress in certain innovations. This provides an opportunity for community-based pharmacists practitioners to learn from practice innovations in other countries. In 2016, the International Pharmaceutical Federation published a report on the global impact of pharmacy-based immunization [96]. Pharmacists in Australia, Canada, England, Netherlands, and Scotland have made substantial progress in expanding the role of the community-based pharmacist [2]. Examples of services related to medication optimization include providing emergency refills, renewing or extending prescriptions, changing drug dosage or formulation, and making therapeutic substitution [2]. Furthermore, in Canada and the United Kingdom, community-based pharmacists are offering comprehensive minor ailment services [97,98]. New Zealand has an opportunity to expand community-based pharmacy services through a new funding model [99]. Changes to qualifications of pharmacists in South Africa which will include prescriptive authority will facilitate expansion of community-based pharmacy services [100]. The United Arab Emirates is implementing initiatives to allow expansion of the role of the community pharmacist; however, other countries in the Middle East continue to struggle with practice expansion [101]. The expansion of the role of the community-based pharmacist has been inconsistent in Asia, but emerging with health assessment, health promotion and medication use reviews [102]. The Pharmaceutical Group of European Union (PGEU) issued a 2030 vision for community-based pharmacy in Europe including expanding pharmacy services to increase access and optimize medication use as part of collaborating primary care team, integrating digital health solutions in practice, showing leadership in personalized medicine, reducing the burden of chronic disease through wellness and prevention and education, identifying public health threats, and providing innovative and effective services to reduce the burden on other services [103]. These components of practice expansion are similar to innovations reported in this manuscript for the US community-based pharmacy practice.

## 5. Conclusions

Community-based pharmacist practitioners in the US are developing and delivering services that meet the needs of patients within the communities they serve. However, there are challenges to overcome but which may be accomplished through policy change, education and training, collaboration, and technology. Through the engagement of community-based pharmacist practitioners, patients will have additional access to more than 180,000 community-based pharmacists, substantially increasing provider capacity, improving care, and reducing overall health care costs to the US health care system.

## Figures and Tables

**Table 1:** Community-Based Patient Care Services.

Medication Optimization	Wellness and Prevention	Chronic Care Management	Acute Care Management	Patient Education	Other Patient Care Services
Medication Packing Home Delivery Medication Reconciliation Appointment-Based Medication Synchronization Medication Adherence Programs Comprehensive Medication Management Services Targeted Medication Review Medication Administration Deprescribing	**Screenings**	Diabetes HTN Cholesterol Asthma Anticoagulation Heart failure Hepatitis C Menopause Monitoring through laboratory testing BHRT saliva testing Anticoagulation A1c TSH Pharmacogenetics Liver function	Test and treat (rapid diagnostics) Influenza Strep H. pylori Urgent care (minor ailments) Triage and referral	Store Brochures Videos Shelf-talkers Posters Individual Group Exercise classes Diabetes prevention program Diabetes education program	Tuberculosis testing Telehealth Durable medical equipment Care transitions Population health Emergency preparedness
	Blood pressure Diabetes Cholesterol Osteoporosis Body Fat HIV Hepatitis C Allergy Lead poisoning				
	**Risk Assessment**				
	Falls Depression Asthma Cardiovascular risk				
	Weight management Tobacco cessation Contraception Bioidentical hormone replacement Fluoride treatments Naloxone Needle exchange Drug take back Nutraceuticals Annual wellness In physician office Pharmacogenomics Sleep assessment Falls prevention Immunizations Pre-travel health services				

BHRT—biodentical hormone replacement therapy.
